# Supplementary material for: Rapid pathogen identification and antimicrobial susceptibility testing in in vitro endophthalmitis with matrix assisted laser desorption-ionization Time-of-Flight Mass Spectrometry and VITEK 2 without prior culture
Source: PLoS One. 2019 Dec 30;14(12):e0227071. doi: 10.1371/journal.pone.0227071 (PMC6936829; doi:10.1371/journal.pone.0227071)
Supplement: Attachment — Submitted filename: Response to Reviewers.docx [file pone.0227071.s001.docx]

**Journal Requirements:**

1. When submitting your revision, we need you to address these additional requirements.

Please ensure that your manuscript meets PLOS ONE's style requirements, including those for file naming. The PLOS ONE style templates can be found at
<http://www.journals.plos.org/plosone/s/file?id=wjVg/PLOSOne_formatting_sample_main_body.pdf> and <http://www.journals.plos.org/plosone/s/file?id=ba62/PLOSOne_formatting_sample_title_authors_affiliations.pdf>

2. We note that you have stated that you will provide repository information for your data at acceptance. Should your manuscript be accepted for publication, we will hold it until you provide the relevant accession numbers or DOIs necessary to access your data. If you wish to make changes to your Data Availability statement, please describe these changes in your cover letter and we will update your Data Availability statement to reflect the information you provide.

**Comments to the Author**

1. Is the manuscript technically sound, and do the data support the conclusions?

The manuscript must describe a technically sound piece of scientific research with data that supports the conclusions. Experiments must have been conducted rigorously, with appropriate controls, replication, and sample sizes. The conclusions must be drawn appropriately based on the data presented.

Reviewer #1: Yes

Reviewer #2: Yes

2. Has the statistical analysis been performed appropriately and rigorously?

Reviewer #1: Yes

Reviewer #2: Yes

3. Have the authors made all data underlying the findings in their manuscript fully available?

The [PLOS Data policy](http://www.plosone.org/static/policies.action#sharing) requires authors to make all data underlying the findings described in their manuscript fully available without restriction, with rare exception (please refer to the Data Availability Statement in the manuscript PDF file). The data should be provided as part of the manuscript or its supporting information, or deposited to a public repository. For example, in addition to summary statistics, the data points behind means, medians and variance measures should be available. If there are restrictions on publicly sharing data—e.g. participant privacy or use of data from a third party—those must be specified.

Reviewer #1: Yes

Reviewer #2: Yes

4. Is the manuscript presented in an intelligible fashion and written in standard English?

PLOS ONE does not copyedit accepted manuscripts, so the language in submitted articles must be clear, correct, and unambiguous. Any typographical or grammatical errors should be corrected at revision, so please note any specific errors here.

Reviewer #1: Yes

Reviewer #2: Yes

5. Review Comments to the Author

Please use the space provided to explain your answers to the questions above. You may also include additional comments for the author, including concerns about dual publication, research ethics, or publication ethics. (Please upload your review as an attachment if it exceeds 20,000 characters)

Reviewer #1: Thank you for submitting your research work to PLOS One.

I find this article very interesting. Congratulations on the excellent work of the entire team and on the well-written manuscript. Despite its limitations, I agree that this technology can become a powerful tool for rapid identification of pathogens in patients with endophthalmitis.

There is a typo: p.6 the word Design (currently is DEISGN).

**Thank you; we have edited the typo.**

Reviewer #2: This is a very interesting study but would recommend the following modifications:

1. Please modify the last sentence of the first paragraph of Introduction, which states that 23-35% of eyes affected by endophthalmitis are left with no light perception. This very high rate of NLP visual outcome may be true for bleb-related endophthalmitis (the reference the authors give), but this is not true for other common types of endophthalmitis such as post-cataract endophthalmitis. Visual outcome is highly associated with pathogen; in coagulase-negative endophthalmitis - the major pathogen of post-cataract endophthalmitis -- NLP vision is very rare (<4%).

**Thank you; this sentence has been modified to state:**

**“Depending on the causative pathogen and pathophysiology, infectious endophthalmitis can lead to poor visual outcomes in the affected eye; for example, the incidence of no light perception ranges from 23% to 35% in bleb-related endophthalmitis(3,4).”**

2. Please add a discussion of this study versus a similar one (not listed in Reference list) published in 2017 by Song Z, et al. "Using MALDI-TOF MS to test Staphylococcus aureus-infected vitreous". Mol Vis. 2017; 23: 407–415. The Song study used porcine eyes injected with S. aureus and directly tested the resulting infected vitreous with MALDI-TOF, so is similar in some regards to the present study -- results may in fact be complimentary. Also, the current study mentions a MALDI-TOF result in a patient with culture-negative endophthalmitis (ref 33), and the Song 2017 study mentions results from 2 patients with culture-positive endophthalmitis in which MALDI-TOF and standard culture yielded the same organism identification. This also may be worth noting.

**The discussion has been modified to state:**

**“Another group previously reported on the implementation of MALDI-TOF MS in identifying a strain of *S. aureus* from *in-vitro* and *ex-vivo* models of endophthalmitis with porcine eyes, and in identifying the causative organisms in culture-positive human endophthalmitis samples, (34). However, we aimed to anticipate the extrapolation of our methods to potential clinical scenarios, and we thus optimized our methodology to require relatively minimal steps in sample preparation, clearly delineate the parameters and materials we employed, and help establish a range of minimum time needed for organism identification with MALDI-TOF MS.**

**Although the previously reported findings on the direct analysis of samples with MALDI-TOF MS support our study, a wider breadth of organisms including other bacteria, mycobacteria, fungi, and polymicrobial infections should also be investigated with MALDI-TOF MS.”**

6. PLOS authors have the option to publish the peer review history of their article ([what does this mean?](https://journals.plos.org/plosone/s/editorial-and-peer-review-process#loc-peer-review-history)). If published, this will include your full peer review and any attached files.


If you choose “no”, your identity will remain anonymous but your review may still be made public.


**Do you want your identity to be public for this peer review?** For information about this choice, including consent withdrawal, please see our [Privacy Policy](https://www.plos.org/privacy-policy).

Reviewer #1: No

Reviewer #2: No


[NOTE: If reviewer comments were submitted as an attachment file, they will be attached to this email and accessible via the submission site. Please log into your account, locate the manuscript record, and check for the action link "View Attachments". If this link does not appear, there are no attachment files to be viewed.]


While revising your submission, please upload your figure files to the Preflight Analysis and Conversion Engine (PACE) digital diagnostic tool, <https://pacev2.apexcovantage.com/>. PACE helps ensure that figures meet PLOS requirements. To use PACE, you must first register as a user. Registration is free. Then, login and navigate to the UPLOAD tab, where you will find detailed instructions on how to use the tool. If you encounter any issues or have any questions when using PACE, please email us at [figures@plos.org](mailto:figures@plos.org). Please note that Supporting Information files do not need this step.
